# Seasonal and Spatial Variations of PM_10_ and PM_2.5_ Oxidative Potential in Five Urban and Rural Sites across Lombardia Region, Italy

**DOI:** 10.3390/ijerph19137778

**Published:** 2022-06-24

**Authors:** Maria Chiara Pietrogrande, Giorgia Demaria, Cristina Colombi, Eleonora Cuccia, Umberto Dal Santo

**Affiliations:** 1 Department of Chemical, Pharmaceutical and Agricultural Sciences, University of Ferrara, Via Fossato di Mortara 17/19, 44121 Ferrara, Italy; dmrgrg@unife.it; 2 Environmental Monitoring Sector, Arpa Lombardia, Via Rosellini 17, 20124 Milano, Italy; c.colombi@arpalombardia.it (C.C.); e.cuccia@arpalombardia.it (E.C.); u.dalsanto@arpalombardia.it (U.D.S.)

**Keywords:** PM_10_ and PM_2.5_ particles, oxidative potential, dithiothreitol and ascorbic acid cell-free assays, chemical tracers, Lombardia region, seasonal and spatial variations

## Abstract

Oxidative potential (OP) of particulate matter (PM) is gaining strong interest as a promising health exposure metric. This study investigated OP of a large set of PM_10_ and PM_2.5_ samples collected at five urban and background sites near Milan (Italy), one of the largest and most polluted urban areas in Europe, afflicted with high particle levels. OP responses from two acellular assays, based on ascorbic acid (AA) and dithiothreitol (DTT), were combined with atmospheric detailed composition to examine any possible feature in OP with PM size fraction, spatial and seasonal variations. A general association of volume-normalized OP with PM mass was found; this association may be related to the clear seasonality observed, whereby there was higher OP activity in wintertime at all investigated sites. Univariate correlations were used to link OP with the concentrations of the major chemical markers of vehicular and biomass burning emissions. Of the two assays, AA was particularly sensitive towards transition metals in coarse particles released from vehicular traffic. The results obtained confirm that the responses from the two assays and their relationship with atmospheric pollutants are assay- and location-dependent, and that their combination is therefore helpful to singling out the PM redox-active compounds driving its oxidative properties.

## 1. Introduction

Particulate matter (PM) is one of the most critical pollutants in the atmosphere, as it has been found to be associated with increased human morbidity and mortality, mainly related to cardio-respiratory diseases [1,2,3]. Although the specific mechanisms of PM-induced health effects are still largely unknown, there is increasing consensus that they involve oxidative stress through the generation of excessive reactive oxygen species (ROS) and/or inadequate antioxidant defenses [4,5,6,7,8,9,10]. Thus, the oxidative potential, representing the capacity of PM to oxidize molecules generating ROS, has been suggested as a representative metric of PM toxicity related to oxidative stress [11,12,13,14,15,16,17].

In this study, we investigated PM oxidative properties in the metropolitan area of Milan and its surroundings in the Lombardy region of northern Italy. This area is a pollution hot spot in Western Europe, suffering from persistent air pollution events characterized by high levels of atmospheric particles. The major sources of ambient PM are related to high population density and several industrial activities in the region, resulting in vehicular emissions, domestic biomass burning and industrial emissions. They are combined with the particular topographical and meteorological conditions in the region, facilitating stagnant conditions, especially during fall and winter, which limit the horizontal and vertical dispersion of air pollutants and promote the formation of secondary inorganic and organic aerosols [18,19,20,21,22,23]. Most of the investigated sites are located in the metropolitan area of Milan, a city with 1.4 million inhabitants, which are heavily exposed to the alarming polluted air. Although the chemical composition of PM in Milan has been quite extensively investigated, its particle-induced toxicity has been scarcely explored [24,25] and even less in the surroundings, despite growing interest in the health effects of PM exposure in highly polluted areas.

To assess the PM toxicity, we measured its oxidative potential with two commonly used acellular assays, which have the advantage of faster reading speed, lower price, less controlled environments and suitability for automation, in comparison with the cellular assays [13,16,17,26,27,28]. One assay uses dithiothreitol (DTT) as a proxy of cellular reductants [29,30,31,32,33] and the other uses ascorbic acid (AA) as a chemical surrogate of physiological antioxidants in the respiratory tract lining fluids (RTLFs) [6,9,34,35]. Both assays measure the depletion rate of the target antioxidants DTT or AA, which is defined as the sample oxidative potential, as it is proportional to the generation rate of ROS [11,13,14,15,16,17]. The PM-induced ROS generation has been found to be substantially driven by the redox-active PM components, mainly metals and some organic compounds [13,16,17,27]. As the two assays involve different redox reactions, they capture different reactivity of the same panels of chemicals. The AA assay has been found to respond mostly to the transition metals [14,15,34,35], while the DTT method to be sensitive to both transition metals and organics, including quinones, oxo-aromatic compounds and HULIS [26,29,30,31,32,33].

Complementary to the recent works of Hakimzadeh et al. [23] and Altuwayjiriet et al. [25], the main goal of this study was to estimate the PM toxicity at urban and rural sites in the Lombardia region by assessing the OP of several PM_10_ and PM_2.5_ particles collected at urban locations in the city center which are strongly impacted by vehicular emissions, and in suburban background sites far from direct traffic emissions. The chemical composition of each PM sample was characterized in terms of the main chemical markers of emission sources and secondary processes that impact the atmosphere in the investigated area. The relationship between the OP responses and the PM composition data was investigated to highlight the main contribution of redox-active PM components to its oxidative properties. 

## 2. Materials and Methods

### 2.1. Chemicals and Materials 

Monosodium phosphate (NaH_2_PO_4_) and disodium hydrogen phosphate (Na_2_HPO_4_) were purchased from Fisher Scientific (Rodano, Milan, Italy). They were used to prepare 0.1 M phosphate buffer at pH 7.4 with ultrapure water (resistivity = 18.2 Ωm) obtained in a Milli-Q^®^ IQ 7000 water purification system (Merck KGaA, Darmstadt, Germany). Next, the buffer was eluted through a Chelex^®^ 100 sodium form resin (Bio-Rad, Segrate, Milan, Italy) to remove any metal contamination. 

Solutions of DTT and DTNB (5,5′-dithiobis (2-nitrobenzoic acid) (Sigma Aldrich s.r.l., Milan, Italy) were prepared in phosphate buffer (10 mM), while solutions of L-ascorbic acid sodium salt (AA) (Sigma Aldrich s.r.l., Milan, Italy) were prepared in ultrapure water (10 mM). Aqueous solutions of the reagents are unstable at room temperature and sensible to light, thus they were preserved in amber glass vials in the dark at −20 °C. 

### 2.2. Sampling Sites and Periods

Filter sampling was conducted at five different sites of the ARPA Lombardia Air Quality Network, with particular focus on the different areas in the megacity Milan. 

Milano Pascal is an urban background station located in the eastern side of Milan, the University area called “Città Studi” (Lat 45°28′24.59″ N, Long 9°13′21.00″ E), in a playground about 130 m from the road traffic. As it is one of the Italian Supersites for the Italian Decree DM 29 November 2012 [36], the complete chemical speciation was performed for each daily PM_10_ and PM_2.5_ collected filters.

Milano Senato is an urban traffic station located in the internal ring road, in the northeastern part of the city center (Lat 45°28′13.79″ N, Long 9°11′50.86″ E). As a Special Station for the Italian Decree DM 29 November 2012, the PM_10_ and PM_2.5_ mass concentrations were measured daily; additionally, the complete chemical speciation was performed for each PM_10_ collected filter.

Milano Marche is an urban traffic station located in the northeastern part of the external ring road (Lat 45°29′46.76″ N, Long 9°11′27.43″ E) impacted by heavy traffic. From 11 April 2019 to 1 April 2020, the PM_10_ and PM_2.5_ mass concentrations were measured daily; additionally, the complete chemical speciation was performed for each PM_10_ collected filter.

Brescia, Villaggio Sereno, is an urban background station located in the south western part of the city of Brescia, in a peripheral area called “Villaggio Sereno” (Lat 45°30′46.92″ N, Long 10°11′31.15″ E).

Schivenoglia is a rural background station, located in the southeastern part of the region, far from specific pollution sources (Lat 45°1′0.67″ N, Long 11°4′34.14″ E). Since this area is not directly impacted by human pressure, it is environmentally relevant to providing information on the physic-chemical phenomena of the atmosphere in a well-characterized area of the Po Valley, improving the knowledge of large-scale processes. Being one of the Italian Supersites for the Italian Decrees, the complete chemical speciation was performed for each daily PM_10_ and PM_2.5_ collected filter.

A total of 357 PM_10_ and PM_2.5_ filters were collected during four monitoring campaigns in winter and spring/summer, as described in detail in Table 1. All sampled filters were analyzed for their PM mass and oxidative potential. The PM_10_ particles were also analyzed for their chemical composition in order to point out spatial (MI_Senato and MI_Pascal in winter, MI_Senato, Brescia and MI_Marche in SS) and seasonal variation (MI_Senato). In addition, PM_2.5_ sampling campaigns were properly carried out for a specific investigation of seasonal variation (MI_Pascal and Schivenoglia) and particle size distribution (PM_10_ and PM_2.5_ at MI_Pascal in winter).

### 2.3. Sampling

Daily PM_10_ and PM_2.5_ particles were sampled on Teflon (Pall) and quartz microfiber (Pall) filters (47 mm diameter). The UNI-EN12341 [37] and US-EPA (CFR40 part. 50 app. J) [38] gravimetric procedures were used, based on different sampling flux of 2.3 m^3^ h^−1^ and 1 m^3^ h^−1^, depending on the environmental conditions. In particular, lower flux is commonly used in winter season to avoid filter loading saturation, especially at high concentration levels [18,19,21,22,23]. The PM mass concentration was determined by gravimetric method on Teflon filters, at 50% relative humidity and 20° C, with a certified precision micro balance with a readability of 1 µg.

The samples were protected against light and temperature between the sampling and the analysis and were stored at 4 °C until analysis.

### 2.4. Chemical Characterization

PM chemical analyses were performed in the laboratories of the Environmental Monitoring Sector, ARPA Lombardia. For each campaign day, quartz and Teflon filters were simultaneously sampled and analyzed in parallel to quantify 39 analytes.

The elemental composition of PM was determined on samples collected on Teflon membranes by energy dispersive X-ray fluorescence (ED-XRF). An Epsilon 4 spectrometer was used from Malvern Panalytical (Monza, Italy). Four measuring conditions were chosen to optimize the sensitivity for the 17 analytes, i.e., Al, Si, P, S, Cl, K, Ca, Ti, V, Cr, Mn, Fe, Ni, Cu, Zn, Br and Pb. 

The EC and OC mass concentrations were measured on a 1.5 cm^2^ punch from PM_10_ quartz filters with the TOT/TOR technique using a SUNSET EC/OC instrument (Sunset Laboratory Inc., Tigard, OR), according to the NIOSH-like and EUSAAR-2 protocols [39].

After extraction with ultrapure water for 30 min in an ultrasonic bath, concentrations of anions, cations and sugars were quantified on another 1.5 cm^2^ punch of each quartz filter. The major ionic species (i.e., Na^+^, NH_4_^+^, K^+^, Mg^2+^, Ca^2+^, Cl^−^, SO_4_^2−^, NO_3_^−^) in the samples were determined by ion-chromatography, IC, using a Compax IC (Metrohm, Origgio, Varese, Italy). Anions were determined on an A supp 5 column (Metrohm) eluting with a 3.2 mM Na_2_CO_3_ +1 mM NaHCO_3_ solution at 0.9 mL min^−1^ flow rate, while cations were measured on a C4 column (Metrhom) eluting with 20 mM nitric acid at 0.6 mL min^−1^ flow rate. All ions were detected with a conductivity detector equipped with a suppression system. Anhydrous sugars were determined on a Carb2 column (Metrhom) eluting with a 3.5 mM NaOH solution at 0.6 mL min^−1^ flow rate and using an amperometric detector.

The last 1.5 cm^2^ punch was used to determine 8 PAHs, namely B(a)P, B(a)A, B(b)F, B(j)F, B(k)F, I(1,2,3,c,d)P, dB(a,h)A and B(e)P). They were measured with a minimum three-day frequency by high pressure liquid chromatography (HPLC, method ISO16362/2005) [40] or gas chromatography with mass spectrometry detector (GC-MS, method ISO12884/2000) [41], with preliminary inter-calibration. Typical minimum detection limits are 0.10 ng m^−3^ for HPLC and 0.05 ng m^−3^ for GC-MS. 

### 2.5. Assessment of the PM Oxidative Potential

OP was quantified using the DTT and AA assays, following the procedure described elsewhere [28,42,43]. Briefly, a quarter of each sampled filter was extracted for 15 min in an ultrasonic bath using 10 mL of a phosphate buffer (0.1 M at pH 7.4), which represents the cell environment to extract the bioavailable components. The extract was filtered on a regenerate cellulose syringe filter (13 mm, 0.22 µm, Kinesis, Redland Bay, Australia) to remove the suspended solid particles. Next, each assay was performed on 3 mL of the solution under the biological relevant temperature of 37 °C (kept constant in a dry bath) and pH of 7.4.

The depletion rate of DTT and AA was measured by using a UV-Vis spectrophotometer (Jasco V-730, Jasco Europe s.r.l., Lecco, Italy) with a 1 cm path length optical cell. 

In the DTT assay, 30 µL of the 10 mM DTT solution was added to the sample (at time zero). At defined times, a 0.50 mL aliquot of the reaction mixture was removed and the reaction was stopped with trichloroacetic acid (0.50 mL of 10%). Then, 50 μL of a DTNB solution (10 mM concentration in phosphate buffer at pH 7.4) was added to each aliquot, generating the DTT-disulphide and 2-nitro-5-thiobenzoic acid (TNB). After waiting two minutes for the reaction to reach completeness, the pH was increased to a value of 8.9 by adding 2.0 mL of Tris-HCl buffer (0.40 M at pH 8.9 with 20 mM of EDTA) to form the mercaptide ion (TNB^2−^), which was spectrophotometrically quantified at 412 nm using a polystyrene cell.

In the AA assay, 30 µL of the 10 mM AA solution was added to the sample (at time zero). Next, the rate of AA depletion (OP^AA^) was directly followed in the spectrophotometric quartz cuvette by measuring the absorption of the ascorbate ion at 265 nm (ε = 14,500 M^−1^ cm^−1^ at pH 7.4) at defined time intervals.

The rate of DTT or AA depletion (nmol min^−1^), defined as the OP response [11,13,14,15,16,17], was determined by linearly fitting the experimental points of the reagent concentration versus time (5, 10, 15, 25, 40 min) [42]. The response of blank filters was determined and subtracted from the data of real PM samples. The obtained OP responses were normalized to the volume of the sampled air to obtain exposure metrics accounting for inhaled air (OP_V_, nmol min^−1^ m^−3^), and to the mass of the sampled particles to compute a parameter describing the PM intrinsic oxidative properties (OP_m_, nmol min^−1^ µg^−1^).

### 2.6. Statistical Analysistion

Student’s *t*-test was conducted to check statistically significant differences (*p* < 0.05) in OP responses and tracer concentrations between two sampling sites or seasons. Moreover, univariate analysis was applied by computing the Pearson’s correlation coefficients (*r*) to investigate the association of the OP responses with the PM_10_ chemical components.

## 3. Results

### 3.1. Overview of Measured PM Oxidative Potential Responses

In order to compare the results of different particle sizes, seasons and sites, Table 2 reports the volume- and mass-normalized OP_V_ and OP_m_ responses from both DTT and AA assays, as well as the PM_10_ and PM_2.5_ mass concentration, expressed as mean and standard deviation values computed for each measurement campaign. The mean PM mass concentration showed a large seasonal variation, with nearly double values in winter (~60 µg m^−3^ and ~45 µg m^−3^ for PM_10_ and PM_2.5_, respectively) compared with those in spring/summer (~30 µg m^−3^ and ~14 µg m^−3^ for PM_10_ and PM_2.5_, respectively). These data confirmed the typical seasonality found across the Lombardia region, where high PM mass levels have been widely reported during the cold period, attributed to the enhanced local emissions (heating and traffic) and lower mixing rate, which lead to the accumulation of pollutants within the mixing layer, with a contribution of ammonium nitrate up to 50% on PM_10_ and even more on PM_2.5_ [18,19,20,21,22,23,24,25,28].

**Figure 1:**
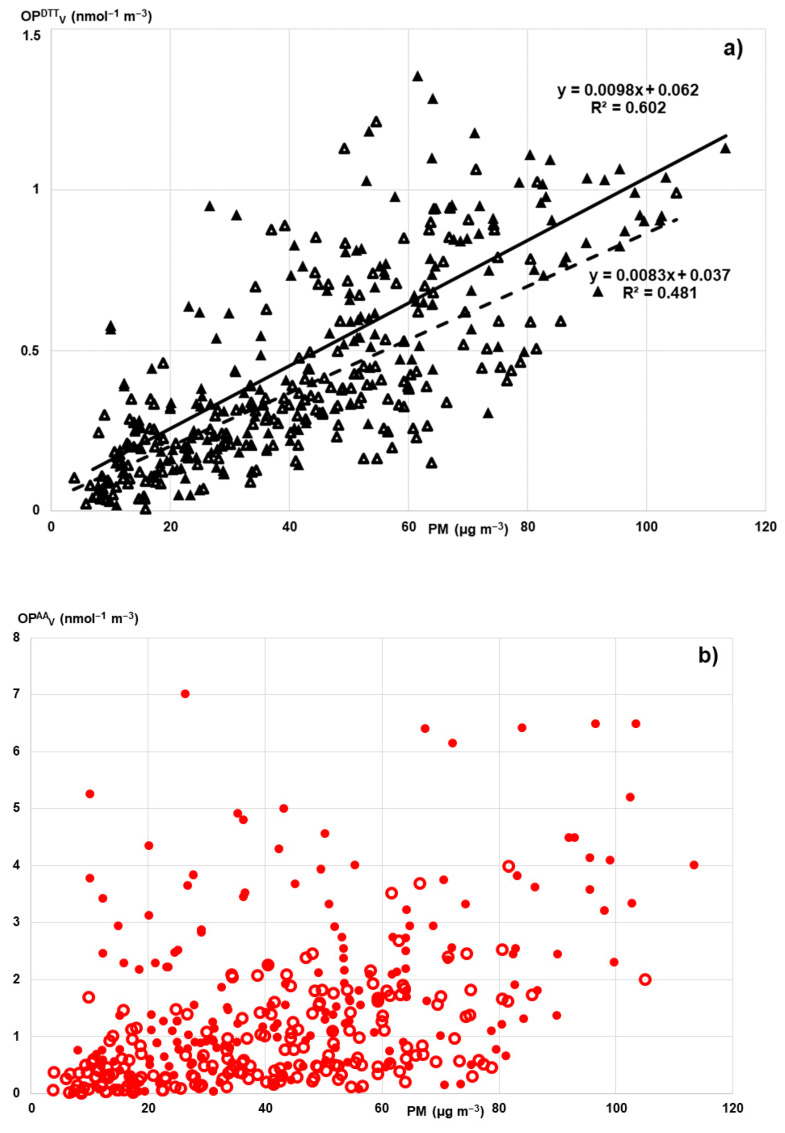
Relationship of the volume-normalized OP^DTT^_V_ and OP^AA^_V_ activity with PM mass concentration of a total of 357 filters collected at five sites across Lombardia region: (**a**) dependence of the OP^DTT^_V_ activity on the PM_10_ (full black triangles) and PM_2.5_ mass (empty black triangles); (**b**) dependence of the OP^AA^_V_ activity on the PM_10_ (full red circles) and PM_2.5_ mass (empty red circles).

To give a general insight into the variability of the PM oxidative properties, all the measured volume-normalized OP^DTT^_V_ and OP^AA^_V_ responses are depicted in Figure 1a,b, respectively, as a function of the PM mass concentration, including both PM_10_ and PM_2.5_ samples (full and empty symbols, respectively).

First of all, it is visually evident that of the two OP metrics, the OP^DTT^_V_ responses were more homogenous (black triangles in Figure 1a), in comparison with the OP^AA^_V_ values, which provided higher discrimination between the samples related with the PM size and sampling site and season (red points in Figure 1b). Figure 1 also reveals that the major variations of OP_V_ activity may be explained by the change in PM mass concentration, as supported by the significant Pearson’s correlation (*p* < 0.001) between OP_V_ and PM mass found for the whole study dataset (Table 3). It is noteworthy that such an association of OP_V_ activity with PM mass concentration is general, regardless of the sampling site and season and also the PM size fraction. Furthermore, the OP^DTT^_V_ responses showed a discrete linear correlation with PM mass, better for PM_10_ samples (R^2^ = 0.602, *p* < 0.001, full line in Figure 1a) than for PM_2.5_ particles (R^2^ = 0.481, *p* < 0.001, dashed line). Consistently, the intrinsic OP^DTT^_m_ values were nearly constant, ranging from 0.007 to 0.013 nmol min^−1^ μg^−1^ (Table 2) throughout the investigation campaigns. This suggest that all the investigated PMs share common sources of OP^DTT^, regardless of urban/rural site, winter/SS season and PM_10_/PM_2.5_ fraction. Similar association of OP^DTT^_V_ values with PM_10_ or PM_2.5_ mass was also observed by separately analyzing the data of each monitoring campaign, as Pearson’s correlation coefficients were mostly found significant at *p* < 0.001 (Table 3).

We can observe largely scattered points in the Figure 1b, although there is a general pattern of higher values for the larger particles (full circles) compared with the fine PM (empty circles). The correlation analysis consistently showed that the association of OP^AA^_V_ with PM mass is poor and even missing for each individual campaign for both PM fractions (Table 3). Accordingly, the measured mass-related OP^AA^_m_ responses displayed variation across the sites, season and particle sizes. 

Within such a variability, we can observe a general trend between the two OP assay responses for each sampling campaign, with higher OP^AA^_V_ values compared with OP^DTT^_V_, i.e., OP^AA^_V_ mean values of 2.22 ± 1.38 nmol min^−1^ m^−3^ (PM_10_ at MI_Senato in winter) and 1.77 ± 1.14 nmol min^−1^ m^−3^ (PM_2.5_ at MI_Marche in winter), in comparison with OP^DTT^_V_ of 0.72 ± 0.28 nmol min^−1^ m^−3^ (PM_10_ at MI_Senato in winter) and 0.62 ± 0.15 nmol min^−1^ m^−3^ (PM_2.5_ at MI_Senato in winter) for the two PM sizes, respectively (Table 2).

Despite the different values, the measured OP^DTT^_V_ and OP^AA^_V_ responses were significantly (*p* < 0.05) inter-correlated by considering the whole dataset, as well as values of each monitoring campaign; this was even better (*p* < 0.01) for PM_10_ than for PM_2.5_, as shown by results of the Pearson’s correlation analysis (Table 3). This is in agreement with our pervious results [28,43,44] and with others from the literature, that reported similar pattern of the two assays [13,17,27,45,46].

### 3.2. Seasonal and Spatial Variation of PM_10_ and PM_2.5_ OP^DTT^ and OP^AA^ Responses

In order to highlight seasonal and spatial variation among the OP^DTT^ and OP^AA^ responses, a two-tail *t*-test was applied to mean values for each sampling campaign to identify significant differences (at *p* < 0.05 level). In particular, in order to investigate the seasonal variation of PM oxidative properties, both sampling campaigns were performed in winter (January–February 2020) and spring/summer (April, June 2019) at the Milan Senato and Pascal sites and also at the rural Schivenoglia site. By comparing the obtained results, we can observe statistically significant (at *p* < 0.05 level) differences between W and SS mean OP values for PM_10_ OP^DTT^_V_ at MI_Senato and for both PM_2.5_ OP^DTT^_V_ and OP^AA^_V_ at MI_Pascal and Schivenoglia (marked by **†** in Table 2). The ratios between the OP_V_ values of the two seasons were computed. For the PM_10_ filters collected at MI_Senato, the cold to warm ratio was 2.3 for OP^DTT^_V_ and 1.3 for OP^AA^_V_. It must be underlined that the W/SS ratio for the PM_10_ concentration was 2.5, which is very close to the value of OP^DTT^_V_ and nearly double of that of OP^AA^_V_. Thus, we can observe nearly double OP^AA^_m_ values in SS compared with those in winter. A similar trend was observed for the PM_2.5_ filters collected at the Pascal site. Here, the cold to warm ratio was 3.0 for OP^DTT^_V_, close to the ratio of 3.5, for PM_2.5_ mass and higher than the ratio of 2.3 for OP^AA^_V_. Otherwise, a different trend was observed at the remote Schivenoglia site, where similar W/SS ratios were computed for OP^DTT^_V_ and OP^AA^_V_, i.e., 5.3 and 5.6, respectively, which were nearly double of that of the 2.7 obtained for the PM_2.5_ mass. 

In order to investigate the impact of urban emissions on PM oxidative properties of the sites other than those inside the city of Milan, the urban background Brescia and the rural background Schivenoglia locations were investigated during each season for PM_10_ and PM_2.5_ fractions, respectively. In general, a substantial homogeneity was observed among the sites for both OP^DTT^ and OP^AA^ responses, likely associated with the wide variability within each monitoring campaign. An exception is OP^AA^_m_, which showed significantly (at *p* < 0.05 level) higher values at the traffic sites compared with the others, i.e., for PM_10_ during SS at Senato and Marche compared with background Brescia, for PM_2.5_ during winter at Marche higher than at Pascal and Schivenoglia, and during SS at Pascal higher than at Schivenoglia. At this last site, significant lower values were also observed for OP^AA^_V_ response in winter and for both OP^DTT^_V_ and OP^AA^_V_ values in SS (indicated by * in Table 2). This may be explained by different PM_2.5_ emission sources at this rural background site, which is not directly impacted by vehicular or other emissions compared with the two Milan Pascal and Marche sites.

### 3.3. Concentrations of PM_10_ Chemical Components

The concentrations of 39 chemical markers were quantified in each daily PM_10_ samples: they comprised major inorganic ions, a total of 17 major and trace elements and carbonaceous components, i.e., organic carbon (OC), elemental carbon (EC), carbohydrates including levoglucosan, a tracer of wood combustion, a total of 8 polycyclic aromatic hydrocarbons (PAHs). The means and standard deviation values of the measured concentrations were computed for each sampling campaign at MI_Senato, MI_Pascal, MI_Marche and Brescia in two periods of the year (Table 4). Student’s *t*-test was applied (at the significance level α = 0.05) to single out significant differences between the sites and the seasons in order to give deeper insight into temporal and spatial variation in PM composition. Overall, the most abundant species (concentration mean ≥ 4 µg m^−3^) were ions NO_3_^−^, SO_4_^2−^ and NH_4_^+^, and OC, followed by EC, Ca, S, Fe, (concentration mean ≥ 1 µg m^−3^) and other organic and inorganic components (at concentration levels around 1 µg m^−3^ or less), i.e., levoglucosan, total aromatic polycyclic hydrocarbons (ΣPAHs), K, Cl, Al and Si.

Concentrations of most of these markers increased in winter compared with the warm season, following the seasonal trend of PM_10_ mass level. It is noteworthy that all the measured values are inside the concentrations range observed in PM_10_ samples of urban and industrial areas in Italy [10,19,22,23,47,48] and in heavy traffic sites all over Europe [4,13,20,49,50]. The cold period in Europe currently displays an increased OC and EC content from traffic and biomass burning sources, which are both also direct emitters of PAH [24,25,39]. A detailed comparison of winter and SS data was performed at the MI_Senato site. Most of the determined components showed a significant increase in winter, namely the NO_3_^−^, NH_4_^+^, K^+^ and Ca^2+^ ions, and carbon components (OC, EC), levoglucosan and ΣPAHs, as well as most of the heavy metals, i.e., Cr, Mn, Fe, Cu, Zn and Pb (marked by **†** in Table 4). 

Furthermore, some measured markers showed significant variation across locations, reflecting the unique composition and emission sources of PM_10_ at each site. In particular, in winter, higher concentrations of Mn, Fe, Zn and Pb were observed at MI_Senato than at MI_Pascal (marked by * in Table 3). These higher concentrations may be related to a larger impact of vehicle emissions, which generates transition metals in both exhaust and non-exhaust particles. In the warm season, more homogeneous values were measured across the three investigated sites, with the exception of some ions (NO_3_^−^, Na^+^ and NH_4_^+^), levoglucosan, total PAHs and sulfur, that showed higher values (*p* < 0.05) at MI_Marche site than at MI_Senato and Brescia. In particular, higher levels of PAHs and sulfur may be related to fugitive dust from traffic [45,50,51,52,53], as MI_Marche site is adjacent to the Milan ring road which is most impacted by heavy duty truck traffic.

### 3.4. Association of PM_10_ Oxidative Potential with Chemical Components

Both OP^DTT^_V_ and OP^AA^_V_ responses were correlated with the concentrations of the measured species in order to highlight significant association. Of the investigated markers, only metals and quinones are redox species which have been found to be reactive towards the OP assays, while the others are correlated or inter-correlated with the main markers of emission sources and secondary processes in the atmosphere. Thus, the relationship among the whole dataset may give a comprehensive insight into the contribution of the main PM processes that influence the PM oxidative properties in the investigated area. Pearson’s correlation analysis was applied by choosing *p* values < 0.05 as statistically significant (in bold in Table 5). As shown in the table, in winter both OP^DTT^_V_ and OP^AA^_V_ responses were significantly correlated with carbonaceous components, mainly OC, EC and anhydrosugars (only at MI_Pascal); with some inorganic species such as ions, i.e., NH_4_^+^ (only OP^DTT^_V_), K^+^, Ca^2+^ and Mg^2+^ (only OP^AA^_V_); and with metals, namely Cr, Mn, Fe, Cu, Zn and Pb, mainly at MI_Pascal. In SS, the OP^AA^_V_ responses at MI_Senato and MI_Marche sites were significantly associated with a wide range of inorganic components, such as ions Ca^2+^ and Mg^2+^, crustal material, i.e., Al, Si, K and Ca, and transition metals such as Ti, V, Mn and Fe. Similar associations were also found for OP^DTT^_V_ at MI_Marche. Among the analytes associated with OP_V_, several species have been identified as markers of vehicular sources, namely the redox-active metals -Cr, Mn, Fe, Cu, Zn and Pb- emitted from motor oil combustion and mechanically generated from tire/brake wear mineral and fugitive re-suspended road dust, and also crustal material such as Al, Si, K and Ca [6,10,14,20,27,30,45,50,51,52,53]. OC and levoglucosan have been commonly used as marker of biomass burning emissions [4,22,39].

### 3.5. Comparisons between OP of PM_2.5_ and PM_10_ Samples

Finally, the oxidative properties of the two particle fractions PM_10_ and PM_2.5_ were compared. A general inspection of the results showed that the OP_V_ responses for fine PM were lower than those for PM_10_. This was expected, since the fine fraction is a part of the total PM_10_ (Table 2). With the aim of investigating the size distribution of PM oxidation capacity in detail, two sampling campaigns were specifically performed at MI_Pascal site in winter to collect the PM_10_ and PM_2.5_ filters simultaneously (Table 1). The mean mass concentrations of PM_10_ and PM_2.5_ were 54 ± 17 µg m^−3^ and 47 ± 17 µg m^−3^, respectively, showing the prevalent contribution of the fine particles, which accounted for 85 ± 17% of the PM_10_ mass. The volume-normalized OP^DTT^_V_ responses were found very similar for both particle-sizes, i.e., 0.65 ± 0.27 nmol min^−1^ m^−3^ and 0.62 ± 0.15 nmol min^−1^ m^−3^ for PM_10_ and PM_2.5_, respectively. Given the dominant contribution of PM_2.5_ to the total PM_10_, intrinsic OP^DTT^_m_ values showed nearly the same values for both particle sizes, with a mean value of 0.012 ± 0.002 nmol min^−1^µg^−1^. Otherwise, the OP^AA^ responses were nearly double for PM_10_ than for PM_2.5_ fractions in terms of both volume- and mass-normalized metrics, namely 2.08 ± 1.69 nmol min^−1^ m^−3^ vs. 1.08 ± 0.62 nmol min^−1^ m^−3^ for OP^AA^_V_ and 0.042 ± 0.029 nmol min^−1^µg^−1^ vs. 0.029 ± 0.011 nmol min^−1^µg^−1^ for OP^AA^_m_ (Table 2).

## 4. Discussion

The reported results clearly show a large variability of the PM oxidative properties among the urban and rural sites across the Lombardia region, as well as between cold and warm seasons. A possible explanation of such a variability may be identified in the contribution of the PM components on its overall oxidative properties by investigating their association with the PM chemical composition. The strength of this study can be magnified by combining the complementary information retrieved from the two DTT and AA assays, which capture specific sensitivity to individual redox-active species. Discussion is mainly focused on volume-normalized OP_V_ metrics, since it represents the actual population exposure, as more relevant than PM mass normalized OP_m_.

Overall, a general dependence of OP_V_ on PM mass was found, particularly for DTT reactivity (Figure 1a). Thus, the measured responses followed the large seasonal variation of PM mass concentration, with high OP_V_ during winter and low during summer, as already observed by the authors in northern Italy [28,42,43] and reported by other authors in Europe, i.e., Weber [26] and Calas [27] in France, Paraskevopoulou [54] and Velali [55] in Greece, and Szigeti [56] in Hungary. However, contrasting results were observed in a previous study in Milan, with higher OP^DTT^_v_ activity in summer and nearly constant OP^AA^_v_ values throughout the year [24]. 

The results of the correlation analysis of OP_V_ with concentration of chemical tracers (Table 5) indicated that the extrinsic redox activity of PM_10_ was driven by a combination of vehicular and biomass burning emissions during the cold season, but by traffic emissions and to lesser degree primary organic compounds during the warm season. Similar contributions to PM_10_ oxidative potential have been reported in other studies in northern Italy [28] and in Milan, in particular [10,20,23,24]. The association of OP^DTT^_V_ with the combination of several different emission sources, comprehensively accounted by the PM mass, may likely be the reason for its general correlation the PM mass. 

Furthermore, a detailed inspection of Pearson’s coefficients revealed that the OP^AA^_V_ values were significantly correlated with a wide list of inorganic components, including crustal material, i.e., Al, Si, K, Ca, and transition metals, such as Ti, V, Mn, Fe, which are tracers of vehicular emissions, in addition to other anthropogenic sources such as oil combustion and industrial activities [5,48,50,54,55,56,57,58,59,60]. This association may likely explain the large variations among the OP^AA^_V_ responses measured in different locations and seasons; they are related to the changes of the most reactive PM components emitted from sources that are temporally and geographically limited (Figure 1b). The dominating dependence of AA reactivity on specific metals may be the main reason for the higher OP^AA^_V_ responses compared with OP^DTT^_V_, since most of the study sites showed high concentrations of traffic-related metals (e.g., Cu, Fe, Mn) towards which the AA assay is more responsive than the DTT. Such a difference has been found in existing studies of urban areas [1,6,13,16,28,42,46]. Enhanced OP^AA^_V_ responses were consistently measured at the two traffic sites in Milan, Senato and Marche. Meanwhile, lower OP^AA^_V_ values were obtained at Schivenoglia, as a rural background site less impacted by traffic, with very similar OP^AA^_V_ and OP^DTT^_V_ responses, i.e., 0.13±0.14 nmol min^−1^ m^−3^ and 0.10 ± 0.007 nmol min^−1^ m^−3^, respectively (Table 2). 

Another relevant result concerned the variation of the measured OP on the particle size. In the study samples, both the extrinsic OP^AA^_V_ and intrinsic OP^AA^_m_ parameters were found to be nearly double for PM_10_ compared with PM_2.5_ fractions (Table 2). This can be explained by the size distribution of the redox-active components mainly driving the AA response, namely Cr, Cu and Pb, which showed significant association with OP^AA^_V_ (correlation results in Table 5). Since these highly reactive transition metals, along with Mn, Sn, Zn and Fe, were found accumulated in the coarse fraction [20,21,22,27,44,52,53,54,55,56,57,58,59,60], they increased the oxidative activity more in the coarse fraction compared with the fine fraction, both in terms of the intrinsic OP^AA^_m_ and the extrinsic OP^AA^_V_ parameters (Table 2). In contrast, the OP^DTT^_V_ responses were very similar for both PM_10_ and PM_2.5_ (Table 2). A possible explanation is that the several markers associated with the DDT response—mainly EC, OC and sugars—are accumulated in the fine fraction [6,9,23,25,39,45,46,55,60], which was the dominant fraction of the PM_10_ particles (Table 2). This result is consistent with the data found in the literature, which report that the volume- and mass-based OP responses may change with the particle size, differently than with the PM mass [20,21,22,28,52,54,55,56,57,58,59]. 

## 5. Conclusions

The main goal of this study was to evaluate the PM oxidative properties in many sites across the Lombardia region, with a focus on the metropolitan area of Milan given its alarming air quality. For PM_10_ particles, the OP^DTT^_V_ and OP^AA^_V_ responses were associated with the concentrations of the main chemical markers of emission sources and secondary processes. The main PM components driving oxidative properties were found to be some transition metals and organic components from vehicle traffic and biomass burning. The elevated OP^AA^ responses likely reflect the contribution of high activity-specific PM components, mainly transition metals in coarse particles, thus indicating that traffic-related sources mainly contributed to OP^AA^, mostly at the investigated locations largely impacted by traffic. Otherwise, the OP from the DTT assay showed more comprehensive dependency on a wide range of pollutants, particularly major carbonaceous components, and thus correlated with the PM mass concentration and strongly reflected the contribution of seasonally-dependent PM components. However, all results based on the correlations of OP with chemical species are to be considered with caution, since—given the significant covariations of many chemical species (including chemical species not analyzed)—correlation does not mean causation. Indeed, the present study may be a further contribution towards elucidating the different sensitivity of the OP^DTT^ and OP^AA^ responses towards the PM components, which remains an open question.

In conclusion, ambient PM in Milan, particularly PM_10_, is potentially redox-active. Given the complexity of different source contributors, in order to be combined with secondary atmospheric processes, there is still much to be learned about the sources and mechanisms implicated in particle-associated redox activity in this urban environment. The findings of these studies will help to implement source-specific regulatory strategies to mitigate the PM-associated toxicity and to improve the protection of human health from the great pathological stress caused by exposure to air pollution. 

## Figures and Tables

**Table 1:** Description of the studied samples: sampling period, site location, PM size fraction and number of collected filters for each sampling campaign.

Sampling Period	Sampling Site	PM Fraction	Sample Number	Abbreviation
2 January–29 February 2020	Milan_Senato	PM_10_	57	MI_Senato W
	Milan_Pascal	PM_10_	57	MI_Pascal W
	Milan_Pascal	PM_2.5_	41	Milan_Pascal W
	Milan Marche	PM_2.5_	52	MI-Marche W
	Schivenoglia	PM_2.5_	60	Schiv W
20–28 April, 8–16 June 2019	Milan_Senato	PM_10_	18	MI_Senato SS
	Brescia	PM_10_	18	Brescia SS
	Milan_Pascal	PM_2.5_	18	MI_Pascal SS
	Schivenoglia	PM_2.5_	18	Schiv SS
1–28 May 2020	Milan_Marche	PM_10_	18	MI-Marche SS

**Table 2:** PM_10_ and PM_2.5_ mass concentration and OP responses measured at the five sites during the various sampling campaigns: means and standard deviation of each campaign data. Units: PM (µg m^−3^); OP_V_ (nmol min^−1^ m^−3^); OP_m_ (nmol min^−1^ µg^−1^).

PM_10_										
	MI_Senato W		MI_Pascal W		MI_Senato SS		MI_Marche SS		Brescia SS	
	Mean	SD	Mean	SD	Mean	SD	Mean	SD	Mean	SD
PM_10_	**64.16 ^†^**	20.18	54.07	17.20	**25.90 ^†^**	11.07	38.11	21.91	26.87	12.02
OP^DTT^_V_	**0.72 ^†^**	0.28	0.65	0.27	**0.32 ^†^**	0.20	0.36	0.26	0.18	0.12
OP^AA^_V_	2.22	1.38	2.08	1.69	1.73	1.08	1.70	0.80	1.05 *	0.77
OP^DTT^_m_	0.013	0.006	0.012	0.002	0.015	0.01	0.008	0.004	0.007	0.00
OP^AA^_m_	**0.038 ^†^**	0.03	0.042	0.02	0.066 ^†,^*	0.04	0.059 *	0.007	0.040 *	0.04
**PM_2.5_**										
	**MI_Pascal W**		**MI_Marche W**		**Schiv W**		**MI_Pascal SS**		**Schiv SS**	
	Mean	SD	Mean	SD	Mean	SD	Mean	SD	Mean	SD
PM_2.5_	**46.07 ^†^**	16.78	51.62	17.91	**40.21 ^†^**	19.39	**13.04 ^†^**	3.90	**14.98 ^†^**	7.73
OP^DTT^_V_	**0.62 ^†^**	0.15	0.43	0.22	**0.53 ^†^**	0.13	**0.21 ^†^**	0.10	0.10 ^†,^*	0.07
OP^AA^_V_	**1.08 ^†^**	0.32	1.77 *	0.74	0.73 ^†,^*	0.20	0.46 ^†,^*	0.38	0.13 ^†,^*	0.14
OP^DTT^_m_	0.013	0.00	0.008	0.00	0.007	0.00	0.016 *	0.01	0.007 *	0.004
OP^AA^_m_	0.029 *	0.01	0.044 *	0.06	0.020 *	0.01	0.033 *	0.02	0.011 *	0.008

**Values in bold** indicate **means** with significant differences (Student’s *t*-test, *p* < 0.05) between winter and SS data (**†**) and between sites (*).

**Table 3:** Dependence of PM oxidative properties on the PM mass concentration and the type of the OP assay: Pearson’s correlation coefficients (*r*) between the OP_V_ responses and the PM mass concentration and between the OP^DTT^_V_ and OP^AA^_V_ responses. Cumulative results on all the study data and results by investigating each sampling campaign, separately. Units: PM (µg m^−3^); OP_V_ (nmol min^−1^ m^−3^).

All Data						
	OP^DTT^_V_			OP^AA^_V_		
PM	0.91 **			0.78 **		
OP^DTT^_V_	1			0.58 **		
**PM_10__Winter**						
	MI_Senato		MI_Pascal			
	OP^DTT^_V_	OP^AA^_V_	OP^DTT^_V_	OP^AA^_V_		
PM_10_	0.42 **	0.30	0.67 **	0.35 *		
OP^DTT^_V_	1	0.47 **	1	0.40 *		
**PM_10__Spring/Summer**						
	MI_Marche		MI_Senato		Brescia	
	OP^DTT^_V_	OP^AA^_V_	OP^DTT^_V_	OP^AA^_V_	OP^DTT^_V_	OP^AA^_V_
PM_10_	0.95 **	0.61 *	0.66 *	0.88 **	0.63 *	0.54
OP^DTT^_V_	1	0.68 *	1	0.71 *	1	0.93 **
**PM_2.5__Winter**						
	MI_Pascal		MI_Marche		Schivenoglia	
	OP^DTT^_V_	OP^AA^_V_	OP^DTT^_V_	OP^AA^_V_	OP^DTT^_V_	OP^AA^_V_
PM_2.5_	0.65 **	0.14	0.69 **	0.35 **	0.79 **	0.40 *
OP^DTT^_V_	1	0.44 **	1	0.27 *	1	0.53 **
**PM_2.5__Spring/Summer**						
	MI_Pascal		Schivenoglia			
	OP^DTT^_V_	OP^AA^_V_	OP^DTT^_V_	OP^AA^_V_		
PM_2.5_	0.40	0.51 *	0.61 **	0.18		
OP^DTT^_V_	1	0.41 *	1	0.36		

* *p* < 0.05 level, ** *p* < 0.001.

**Table 4:** Concentration values of the chemical components of PM_10_ filters collected in each monitoring campaign: means and standard deviation data. Units: all analytes (µg m^−3^); ΣPAHs (ng m^−3^).

	MI_Senato W		MI_Pascal W		MI_Senato SS		MI_Marche SS		Brescia SS	
	Mean	SD	Mean	SD	Mean	SD	Mean	SD	Mean	SD
Cl^−^ (µg m^−^^3^)	0.57	0.45	0.65	0.45	0.40	0.18	0.25	0.29	0.78	0.22
NO_2_^−^ (µg m^−3^)	0.17	0.11	0.04	0.01	0.19	0.12			0.13	0.08
NO_3_^−^ (µg m^−3^)	**16.82 ^†^**	9.08	15.37	8.85	1.77 ^†,^*	1.21	6.97 *	4.51	2.12 *	1.33
SO_4_^2−^ (µg m^−3^)	2.48	1.26	2.51	1.39	2.50	1.23	3.49	4.15	2.91	1.41
Na^+^ (µg m^−3^)	0.55	0.49	0.49	0.29	0.66 *	0.25	3.17 *	2.43	0.60 *	0.27
NH_4_^+^ (µg m^−3^)	**5.16 ^†^**	2.67	4.56	2.50	0.74 ^†,^*	0.43	2.81 *	1.93	0.73 *	0.51
K^+^ (µg m^−3^)	**0.64 ^†^**	0.38	0.46	0.24	**0.20 ^†^**	0.03	0.28	0.11	0.09	0.04
Mg^2+^ (µg m^−3^)	0.17	0.05	0.09	0.03	0.14	0.05	0.34	0.38	0.15	0.06
Ca^2+^ (µg m^−3^)	**1.66 ^†^**	1.24	0.83	0.38	**0.63 ^†^**	0.34	1.71	2.54	0.99	0.58
OC (µg m^−3^)	**10.38 ^†^**	4.23	9.74	3.88	**5.45 ^†^**	1.16	5.91	2.07	4.54	1.16
EC (µg m^−3^)	**1.64 ^†^**	0.90	1.38	0.83	**0.52 ^†^**	0.18	0.67	0.26	0.60	0.13
Mannitol	0.12	0.10	0.03	0.01		0.00				
Levo	**1.10 ^†^**	0.71	0.99	0.73	0.06 ^†,^*	0.01	0.21 *	0.12	0.06 *	0.01
Manno	0.10	0.07	0.11	0.08	0.00	0.00				
Galacto	0.12	0.31	0.07	0.05	0.00	0.00			0.05	0.03
ƩPAHs (ngm^−3^)	**3.71 ^†^**	3.77	2.82	2.22	0.02 ^†,^*	0.05	0.20 *	0.13	0.04 *	0.12
S	1.66	0.80	1.01	0.50	1.14 *	0.55	2.15 *	2.10	1.11 *	0.54
Cl	**1.07 ^†^**	0.70	1.08	0.54	**0.31 ^†^**	0.17	0.28	0.27	0.24	0.31
Al	0.52	0.26	0.34	0.16	0.33	0.23	0.64	0.65	0.52	0.39
Si	1.61	0.72	1.19	0.51	0.96	0.62	1.71	1.53	1.28	0.87
K	**0.74 ^†^**	0.34	0.71	0.31	**0.24 ^†^**	0.11	0.41	0.23	0.27	0.15
Ca	**2.48 ^†^**	1.70	1.17	0.55	**0.84 ^†^**	0.42	2.09	2.56	1.01	0.60
Ti	0.08	0.04	0.05	0.02	0.04	0.02	0.07	0.06	0.04	0.03
V	0.00	0.00	0.00	0.00	0.00	0.00	0.00	0.00	0.00	
Cr	**0.02 ^†^**	0.01	0.02	0.01	**0.01 ^†^**	0.00	0.01	0.00	0.01	0.00
Mn	0.05 ^†,^*	0.02	0.03 *	0.01	**0.02 ^†^**	0.01	0.02	0.01	0.02	0.01
Fe	4.00 ^†,^*	1.72	2.37 *	0.83	**1.05 ^†^**	0.34	1.57	0.92	0.70	0.37
Ni	0.01	0.01	0.01	0.00	0.00	0.00	0.00	0.00	0.00	0.00
Cu	0.14 ^†,^*	0.02	0.11 *	0.03	**0.03 ^†^**	0.01	0.03	0.02	0.02	0.01
Zn	0.40 ^†,^*	0.54	0.12 *	0.05	**0.03 ^†^**	0.01	0.06	0.04	0.06	0.04
Br	0.02	0.01	0.02	0.03	0.01	0.00	0.01	0.00	0.01	0.00
Pb	0.11 ^†,^*	0.15	0.05 *	0.02	**0.01 ^†^**	0.01	0.02	0.01	0.01	0.00

**Values in bold** indicate **means** with significant differences (Student’s *t*-test, *p* < 0.05) between winter and SS data at MI_Senato (**†**) and between sites (*).

**Table 5:** Pearson’s correlation coefficients (*r*) between the OP^DTT^_V_ and OP^AA^_V_ responses and the PM_10_ chemical components in winter campaigns at MI_Senato and MI_Pascal ad spring/summer periods at MI_Senato, MI_Marche and Brescia sites.

	MI_Senato W		MI_Pascal W		MI_Senato SS		MI_Marche SS		Brescia SS	
	OP^AA^_v_	OP^DTT^_v_	OP^AA^_v_	OP^DTT^_v_	OP^AA^_v_	OP^DTT^_v_	OP^AA^_v_	OP^DTT^_v_	OP^AA^_v_	OP^DTT^_v_
Cl^−^ (µg m^−3^)	0.34	0.19	**0.50**	0.32	0.47	0.57	0.34	0.08	−0.01	0.01
NO_2_^−^ (µg)					0.19	**0.87**			−0.17	−0.13
NO_3_^−^ (µg m^−3^)	−0.11	0.11	−0.14	0.42	**0.82**	0.58	0.26	0.47	0.52	0.56
SO_4_^2−^ (µg m^−3^)	0.26	0.20	−0.14	0.08	0.70	0.54	0.56	**0.80**	0.28	0.35
Na^+^ (µg m^−3^)	−0.10	−0.07	−0.16	−0.22	**0.79**	0.62	0.55	**0.95**	−0.01	0.02
NH_4_^+^(µg m^−3^)	0.06	0.20	−0.16	**0.46**	0.58	0.32	0.00	0.13	0.41	0.47
K^+^ (µg m^−3^)	0.03	0.08	**0.47**	0.22	0.00	0.00	−0.12	−0.34	0.18	0.00
Mg^2+^(µg m^−3^)	0.06	0.09	**0.47**	0.17	**0.93**	**0.90**	**0.64**	**0.83**	0.06	−0.02
Ca^2+^(µg m^−3^)	**0.38**	0.29	0.15	0.09	**0.92**	0.67	**0.64**	**0.84**	0.12	0.07
OC (µg m^−3^)	**0.41**	0.35	**0.35**	**0.80**	0.37	0.28	0.26	0.56	0.50	0.71
EC (µg m^−3^)	**0.42**	**0.45**	**0.57**	**0.64**	0.65	0.59	0.46	**0.67**	0.44	0.61
Mannitol	0.02	**0.44**								
Levo	0.33	0.25	**0.51**	**0.78**	0.38	0.67	−0.07	0.46	0.38	0.36
Manno	0.26	0.30	**0.53**	**0.73**						
Galacto	−0.09	0.25	0.13	0.09					0.72	0.66
ƩPAHs	−0.05	0.12	−0.19	0.29	−0.04	−0.42			−0.26	−0.27
S	0.24	0.28	−0.09	0.32	0.54	0.20	0.58	**0.79**	0.23	0.29
Cl	**0.46**	**0.44**	0.21	**0.56**	−0.25	−0.37	0.49	0.27	0.08	0.08
Al	0.15	0.24	−0.04	−0.05	**0.88**	0.44	**0.62**	**0.86**	0.32	0.33
Si	0.22	0.25	0.02	−0.05	**0.78**	0.39	**0.66**	**0.89**	0.30	0.35
K	**0.36**	**0.51**	0.24	**0.80**	**0.79**	0.42	**0.68**	**0.95**	0.40	0.50
Ca	**0.38**	0.31	0.00	−0.07	**0.88**	0.48	**0.65**	**0.85**	0.49	0.54
Ti	0.14	0.23	0.05	0.14	**0.85**	0.39	**0.69**	**0.92**	0.31	0.33
V	0.20	0.10	−0.32	−0.23			**0.65**	**0.87**		
Cr	0.25	0.27	**0.42**	**0.37**	0.21	0.41	0.49	0.50	**0.77**	0.76
Mn	0.29	**0.36**	0.26	**0.37**	**0.76**	0.62	**0.74**	**0.93**	0.58	0.70
Fe	0.30	0.24	0.38	**0.39**	0.70	0.67	**0.68**	**0.80**	0.59	0.66
Ni	0.29	0.27	0.25	0.29	0.65	**0.71**	0.14	−0.52		
Cu	0.33	0.27	**0.49**	**0.46**	0.31	0.58	0.34	0.31	0.10	0.26
Zn	0.24	0.16	0.13	**0.38**	0.66	0.36	0.35	0.30	0.31	0.39
Br	0.19	0.27	0.08	−0.03	0.57	0.23	0.51	**0.67**	0.22	0.29
Pb	0.33	0.26	0.26	**0.73**	−0.03	0.73	0.52	0.39	−0.05	0.15

**Values in bold** indicate significant correlation (at *p* < 0.05 level) between the data.

## Data Availability

The data presented in this study are available on request from the corresponding author.
